# Risk Factors for Recurrent Thrombosis in Patients with Antiphospholipid Syndrome—A Single-Centre Cohort Study

**DOI:** 10.1055/a-2646-9016

**Published:** 2025-07-18

**Authors:** Natali Karandyszowska, Francesca Faustini, Hevgin Alagündüz, Jacob Widaeus, Felicia Carlens, Ann-Louise Jensen, Anna Oksanen, Maria Magnusson, Iva Gunnarsson, Elisabet Svenungsson, Aleksandra Antovic, Maria Bruzelius

**Affiliations:** 1 Department of Medicine Solna, Karolinska Institutet, Stockholm, Sweden; 2 Department of Hematology, Coagulation Unit, Karolinska University Hospital, Stockholm, Sweden; 3 Division of Rheumatology, Department of Medicine Solna, Karolinska Institutet and Rheumatology, Karolinska University Hospital, Stockholm, Sweden; 4 Department of Medicine, Danderyd Hospital, Stockholm, Sweden; 5 Department of Clinical Sciences, Karolinska Institutet, Danderyd Hospital, Stockholm, Sweden; 6 School of Medical Sciences, Örebro University, Örebro, Sweden; 7 Division of Biostatistics, Institute of Environmental Medicine, Karolinska Institutet, Solna, Sweden; 8 Clinical Science, Intervention and Technology (CLINTEC), Karolinska Institutet, Stockholm, Sweden; 9 Department of Molecular Medicine and Surgery, Karolinska Institutet, Stockholm, Sweden

**Keywords:** aGAPSS, antiphospholipid syndrome, recurrent thrombosis, risk factors, thrombocytopenia

## Abstract

**Background:**

Recurrent thrombosis poses a clinical challenge in patients with antiphospholipid syndrome (APS). There are limited data on risk factors due to its rarity.

**Aims:**

This study aimed to study the association between cardiovascular (CV) and APS-related risk factors and recurrent thrombosis and evaluate the adjusted Global Anti-Phospholipid Syndrome Score (aGAPSS).

**Methods:**

This retrospective cohort study comprised APS patients at Karolinska University Hospital, Sweden, from 2014 to 2020 with follow-up until the last medical visit or death. Multiple thrombotic events per patient were included. Cox proportional hazard model estimated hazard ratios (HRs) and 95% confidence intervals (CIs). Logistic regression and Poisson regression were conducted to further examine the relation between risk factors and recurrent thrombosis.

**Results:**

The cohort included 250 patients (67% women and 62% primary APS) with a median age of 44.5 (35–59) years. Forty-nine recurrent thrombotic events occurred in 36 patients, yielding an incidence of 4.46 (95% CI 3.30–5.90) per 100 person-years. Thrombocytopenia was associated with recurrent thrombosis (HR 2.57 [95% CI 1.01–6.02]). Although CV risk factors were not consistently significant for recurrent thrombosis, chronic kidney disease (CKD) indicated an increased probability (OR 2.55 [95% CI 1.01–6.26]). For each point of aGAPSS, the HR for recurrent thrombosis increased by 10% (1.10 [95% CI 1.01–1.19]). Notably, inadequate anticoagulation triggered recurrence in almost a quarter of cases.

**Conclusion:**

Thrombocytopenia was confirmed as a major risk factor for recurrent thrombosis. CKD warrants closer attention in future assessment. Although an increase in aGAPSS was associated with recurrent thrombosis, further evaluation is required. Improving anticoagulation treatment is essential to reduce recurrence.

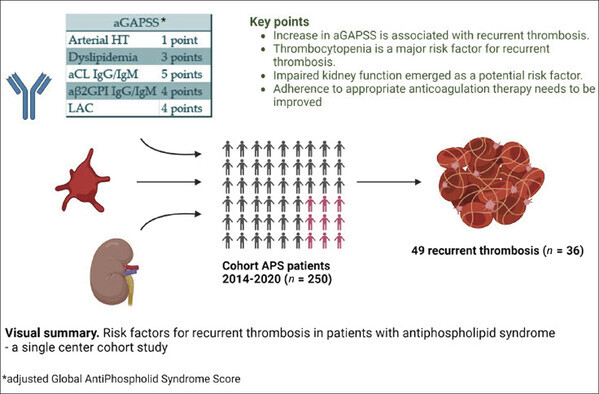

## Introduction

Antiphospholipid syndrome (APS) is a rare but potentially fatal autoimmune condition affecting predominantly a younger population, with a higher prevalence among women. Despite continuous antithrombotic treatment, the risk of new thrombotic events remains high. 1

To date, the estimated incidence and prevalence of APS are low, 1 to 2 cases and 17 to 50 cases per 100,000, respectively. 2 Still, antiphospholipid antibodies (aPLs), that is, cardiolipin (aCL) and/or β _2_ -glycoprotein-1 (aβ _2_ -GP1) antibodies of the IgG and/or IgM isotypes and/or presence of lupus anticoagulant (LA) have been shown to be present in 9.5% of all patients with venous thromboembolisms, 13.5% with ischemic stroke, and 11% with myocardial infarction (MI), possibly indicating that APS is often an underdiagnosed condition. 3 The frequency of recurrent thrombotic events varies depending on the studied APS population; it is estimated to affect one-fifth of all APS patients over a period of 5 years and is associated with increased morbidity and mortality. 4 5 Patients with a high-risk aPL profile at diagnosis, that is, presence of LA, triple aPL positivity, or the presence of persistently high aCL and aβ _2_ -GP1 titers, have an increased risk for APS manifestations, including recurrent thrombosis. 1 The 5-year incidence of recurrent thrombotic events has been reported as high as 26% in patients with triple aPL positivity; however, 19% were not on continuous anticoagulant treatment. 1

APS treatment guidelines recommend regular assessment of cardiovascular (CV) risk factors. 6 7 There is, nevertheless, a significant heterogeneity among studies regarding the impact of traditional CV risk factors on recurrent thrombosis. 8 9 10 The Global Anti-Phospholipid Syndrome Score (GAPSS) was originally constructed for patients with systemic lupus erythematosus (SLE) without previous thrombosis to predict risk for first thrombosis, but was later extrapolated to APS to predict the risk for recurrent events. 11 In the prediction score, hypertension and hyperlipidemia are included together with the patient's aPL profile. The adjusted score, aGAPSS, is associated with an increased risk for thrombotic recurrence, and it is suggested to be a useful tool at annual visits for risk assessment. 12 Yet, there may be additional risk factors that are important for identifying APS patients at risk. For instance, a decline in kidney function is a known risk factor for CV events and is often found in SLE patients with APS. 13 Thrombocytopenia, the most frequent hematological APS manifestation, which occurs in 20 to 50% of the cases, has been linked to recurrent thrombosis. 14 15 16 17 In our Swedish single-center cohort, we aimed to study the association between CV and APS-related risk factors and recurrent thrombosis and to evaluate the adjusted Global Anti-Phospholipid Syndrome Score (aGAPSS).

## Materials and Methods

This was a single-center cohort study based on retrospectively collected data on patients with APS at Karolinska University Hospital (Karolinska) in Sweden, a tertiary referral hospital in Region Stockholm. The project was approved by the Swedish Ethical Review Authority (2020-02333).

### Population

**Fig. 1:**
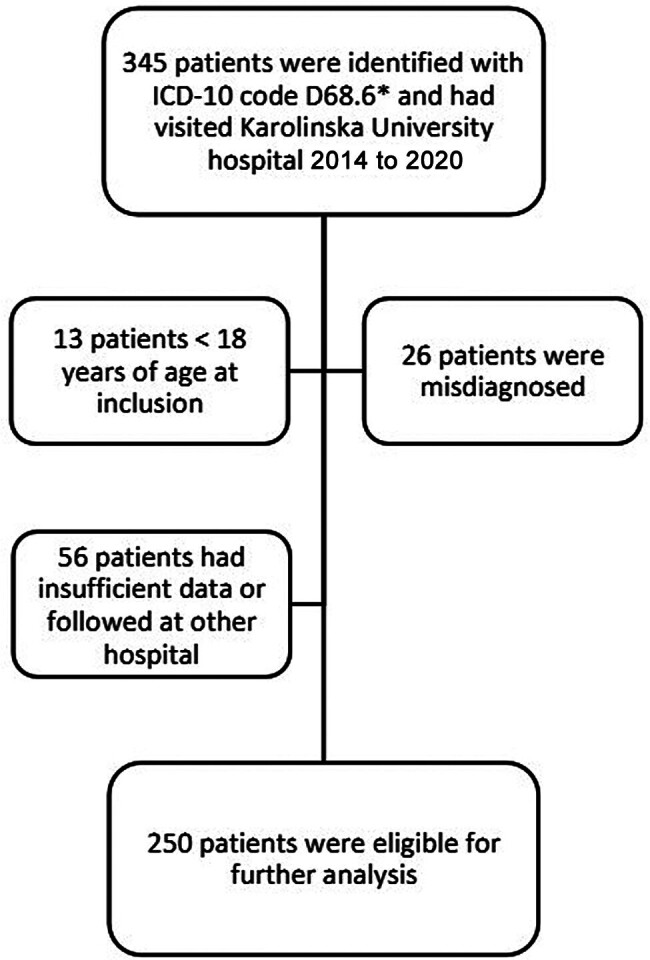
Flowchart describing the cohort and total size after exclusion. The study initially included 345 patients according to ICD-10 coding of APS diagnosis in Karolinska University Hospital, resulting in 250 patients. Causes of exclusion are described in the flow chart. Among these, three patients did not have a follow-up visit within the study period. This was due to the late timing of their inclusion, leaving insufficient time for a subsequent visit to be recorded. These patients were not considered lost to follow-up but were retained in the analysis based on available baseline data. APS, antiphospholipid syndrome.

For inclusion, patients were required to have had at least one visit at the Department of Hematology or Rheumatology, between January 2014 and August 2020 with a registered ICD-10 diagnosis indicative of APS (D68.6*). This included both patients with a current APS and those who developed APS during the study period. Exclusion criteria were age under 18 years, misdiagnosis of APS according to established classification criteria, 18 referral without a medical visit at Karolinska, or insufficient data ( Fig. 1 ).

### Data Collection

Data were retrieved from the electronic medical record system. The main data were collected in conjunction with the first and last documented visit during the study period. If a patient had suffered from multiple recurrent thrombotic events during follow-up, each thrombotic event, along with the time interval between events, was registered. Medical records were reviewed in depth to investigate the conditions surrounding every single thrombotic event and to identify possible triggers. Information regarding antithrombotic treatment, that is, type and dosage, compliance, or a recent switch, in treatment preceding the event was collected. Furthermore, risk factors associated with the recurrent event were documented, including surgery in the prior 30 days, pregnancy, infection, and estrogen-containing contraceptives. Death and cause of death were also registered, if applicable.

### Variables

The outcome variable comprised any thrombotic events that were registered chronologically and followed between January 2014 and August 2020. Arterial thrombotic events (ATEs) included stroke, transient ischemic attack, MI, extremity thrombosis, and other types of arterial thrombosis such as end-organ embolism. Venous thromboembolic events (VTEs) were deep vein thrombosis (DVT) in the lower limbs, pulmonary embolism, or other localizations, that is, arm, splanchnic, or cerebral venous sinus thrombosis.

The collected variables included age, sex, aPL profile, antithrombotic treatment (anticoagulant and antiplatelet drugs), date of thrombotic or obstetric event, date of APS diagnosis, coexisting autoimmune systemic diseases (e.g., SLE and other rheumatological diseases), hematological manifestation with thrombocytopenia defined as platelet count <130 × 10 ^9^ /L 19 and CV risk factors, that is, current smoking, obesity (body mass index over 30 kg/m ^2^ ), hypertension, hyperlipidemia, diabetes mellitus, and chronic kidney disease (CKD) defined as an estimated glomerular filtration rate (eGFR) <60 mL/min/1.73 m ^2^ . Hypertension was defined as the use of antihypertensive medication and/or a diagnosis set by a physician in a free text or found listed with an ICD code in the medical records. Hyperlipidemia and diabetes mellitus were defined accordingly. Current smokers were identified using a free-text search for the term “smoking” in the medical records. We also collected laboratory data on platelet count and kidney function with creatinine and eGFR over time. If the patient had repeated values over a short period of time (hospitalization), the first, the lowest (platelet count), or the highest value (creatinine and its corresponding eGFR), and the last value from that period were registered.

### Detection of Antiphospholipid Antibodies and Categorization by Serological Risk Profile

aCL and aβ _2_ -GP1 were analyzed in serum using multiplexed bead technology (Luminex) with the BioPlex 2200 system (Bio-Rad, Hercules, CA), used at Karolinska University Hospital since 2014. 19 20 The presence of aCL and aβ _2_ -GP1 was defined as titers above 10 U/mL for IgG isotype and above 30 U/mL for IgM isotype, all in the >99th percentile. LA presence was defined according to Scientific and Standarization Committee International Society on Thrombosis and Haemostasis's guidelines. 21 We thoroughly checked if patients were on anticoagulant treatment at the time of aPL testing. Categorization of serological risk profile was as follows 18 :

- Single positivity: Presence of aCL or aβ _2_ -GP1 of IgG or IgM isotype.
- Double positivity: Presence of either two of any of the isotypes of aCL and aβ _2_ -GP1 (not two isotypes of the same aPL) or one of them combined with LA.
- Triple positivity: Presence of aCL and aβ _2_ -GP1 combined with LA.
- Presence of LA.

### The Adjusted Global Anti-Phospholipid Syndrome Score

The aGAPSS was calculated for each patient at first visit, including both CV risk factors and serological profiles as follows:

- Hypertension (1 point)
- Hyperlipidemia (3 points)
- LA and aβ _2_ -GP1 (IgM or IgG) antibodies (4 points)
- aCL (IgM or IgG) antibodies (5 points)

The minimum score is 4 points in the absence of any CV risk factor for any isotype, and the maximum score is 17 points. 11

### Follow-Up Time and Outcome

The start of follow-up was defined as the date of first visit from January 1, 2014. Patients were followed until the last visit documented in the medical records up to August 31, 2020, death, or lost to follow-up. Recurrent thrombosis was defined as a new onset of thrombosis during the follow-up time and must have occurred after the first visit. Patients with only one visit registered in the medical records were registered as a first visit with the assumption of no recurrence during the remaining study period.

### Statistical Methods

Descriptive statistics were used to report the characteristics of the cohort, presenting percentages for categorical variables and medians with interquartile range (IQR) for continuous variables. Differences between groups of patients with recurrent thrombosis and those without recurrence were tested using χ ^2^ test and Fisher's exact test for categorical variables and a *t* -test for numerical variables.

Univariable Cox proportional hazards regression analyses were first performed for each predictor variable, including APS-related risk factors (secondary APS, serological risk profile, and thrombocytopenia), CV risk factors, and aGAPSS, in relation to recurrent thrombosis. Subsequently, multivariable models were constructed for each predictor, adjusted for age and sex. All recurrent thrombotic events were included in the analyses, allowing some patients to contribute with multiple events. These were accounted for using a clustered robust estimator for standard errors. Additionally, time varying Cox models were estimated for platelet count and eGFR for which several measurements were available over time for most individuals. The time intervals between measurements were not consistent across patients, as data were obtained during routine clinical follow-up. This model allowed multiple observations for each person, with time measured from the date of CKD or platelet count measurement until the next measurement, thrombosis, or end of follow-up. These were handled using a clustered robust estimator for the standard errors. Results were presented as hazard ratios (HRs) with 95% confidence intervals (CIs) and corresponding *p* -values.

Incidence rates (IRs) and rate ratios (IRRs) were estimated using Poisson regression, offsetting for time under observation. In addition, a logistic regression model was used. Logistic regression is not able to account for the differences in observation time between patients, which is why the analysis is purely descriptive and cannot be used to draw conclusions. It was added as the sample size and number of events is relatively low and descriptives provide information even when testing is difficult.

For risk stratification, patients were categorized into three serological groups: (1) Single or double aPL positivity, (2) LA positivity only, and (3) triple positivity. Due to the limited number of cases, single and double positivity were combined into one group, which served as the reference group for statistical comparisons. This classification enabled more robust analyses. aGAPSS was used both as a continuous variable and dichotomized into 4 to 8 points and 9 to 17 points. The group with 4 to 8 points was considered at lower risk as it did not include patients with the high-risk serological aPL profile, triple positivity.

Body mass index was omitted from the analysis due to missing data in 25% of patients.

All analyses were performed using R version 4.2.3 (R Core Team, 2022). Cox regression was estimated with the “survival” package. Any *p* -values with <0.05 were considered statistically significant.

## Results

Two hundred and fifty patients with a definitive APS diagnosis were included in the study ( Fig. 1 ). The cohort accumulated 1,339 person-years with a median follow-up of 5 (4–6) years. The median age at inclusion was 44.5 (35–59) years, with a predominance of women (68%), which remained after excluding women with only obstetric APS (58%; Table 1 ). Women were significantly younger at diagnosis compared with men, 37 (28–51) and 48 (39–60) years, respectively. The difference remained significant even after excluding women with only obstetric manifestations. VTE was the most common APS manifestation, documented in 151 (60%) patients, followed by ATE in 89 patients (36%; Table 1 ). Forty-nine recurrent thrombotic events occurred in 36 (14%) patients with an estimated incidence of 3.7 per 100 person-years (95% CI 2.5–5.0). The mean time to first recurrent thrombosis among patients who experienced a recurrence was 2.1 years (95% CI 1.42–2.75). The median age at the time of the first recurrent thrombotic event was 45.5 (37–64) years. Ninety six (38%) patients had secondary APS, and 88 (73%) of these had SLE ( Table 1 ).

**Table 1:** Clinical characteristics and serological antiphospholipid antibody profile

		Thrombotic event during follow-up		
		Yes	No	*p* -Value
	250	36	214		
Age, years (IQR)	44.5 (33–50)	43.0 (34–62)	45.0 (34–58)	0.94
Women (%)	169 (67.6)	24 (66.7)	145 (67.8)	0.75
Disease duration (years; IQR)	0.2 (0–5.8)	1.26 (0–5)	0.08 (0–10)	0.06
Thrombotic events, at first visit (%)				
Any thrombosis	222 (89)	36 (100)	186 (87)	0.15
**Arterial**	89 (36)	18 (50)	71 (33)	0.08
Stroke/TIA	70 (28)	14 (39)	56 (26)	0.17
Myocardial infarction	13 (5)	3 (8)	10 (5)	0.61
**Venous**	151 (60)	20 (56)	131 (61)	0.65
DVT lower limbs	113 (46)	20 (56)	93 (44)	0.24
DVT other sites	19 (8)	3 (8)	16 (8)	1.00
Pulmonary embolism	69 (28)	10 (28)	59 (28)	1.00
**Thrombotic microangiopathy**	36 (14)	6 (19)	30 (14)	0.87
**CAPS**	8 (3)	3 (8)	5 (2)	0.17
**Obstetric**	70 (28)	9 (25)	61 (29)	0.82
Obstetric only	28 (11)	1 (3)	27 (13)	0.15
Serological profile of antiphospholipid antibodies				0.06
Single positive	9 (4)	0	9 (4)	
Double positive	80 (32)	8 (22)	73 (34)	
Triple positive	121 (48)	24 (67)	96 (45)	
Lupus anticoagulant	40 (16)	4 (11)	36 (17)	
Primary APS	154 (62)	16 (44)	138 (65)	0.42
Secondary APS	96 (38)	20 (56)	76 (36)	0.42
SLE	75 (30)	15 (42)	60 (28)	0.17
Platelet count (IQR)	238 (187–291)	267 (190–321)	234 (187–282)	0.30
Thrombocytopenia a (%)	20 (8)	5 (14)	15 (7)	0.43
Cardiovascular risk factors (%)				
Hypertension	103 (41)	18 (50)	85 (40)	0.11
Hyperlipidemia	67 (27)	11 (30)	56 (26)	0.57
Diabetes	12 (5)	3 (8.3)	9 (4)	0.14
Current smoking	31 (12)	7 (19)	24 (11)	0.27
Kidney function, eGFR b (IQR)	79 (68–91)	71 (54–83)	81 (69–91)	<0.01
Chronic kidney disease c	44 (18)	12 (33)	32 (15)	<0.05
aGAPSS, points (IQR)	13 (9–13)	13 (10–14)	12.5 (9–13)	<0.01
Low: 4–8 points (%)	48 (19)	4 (11)	44 (21)	
High: 9–17 points (%)	202 (81)	32 (89)	170 (79)	
**Treatment type** (%)	250	36	214	
Warfarin	71 (28)	13 (36)	58 (27)	0.55
LMWH	58 (23)	5(14)	53 (25)	1
DOAC	15 (5)	3 (8)	9 (4)	1
Platelet inhibitor	80 (32)	10 (27)	70 (33)	0.56
Platelet inhibitor and anticoagulant	39 (16)	6 (16)	33 (16)	1

Abbreviations: aGAPSS, adjusted Global Antiphospholipid Syndrome Score; CAPS, catastrophic antiphospholipid syndrome; DOAC, direct oral anticoagulant; DVT, deep vein thrombosis; IQR, interquartile range; LMWH, low-molecular-weight heparin; SLE, systemic lupus erythematosus; TIA, transient ischemic attack.

a Platelet count <130 × 10 ^9^ /L.

b Estimated glomerular filtration rate (mL/min/1.73 m ^2^ ).

c eGFR <60 mL/min/1.73 m ^2^ .

### Antiphospholipid Syndrome-Related Risk Factors

**Fig. 2:**
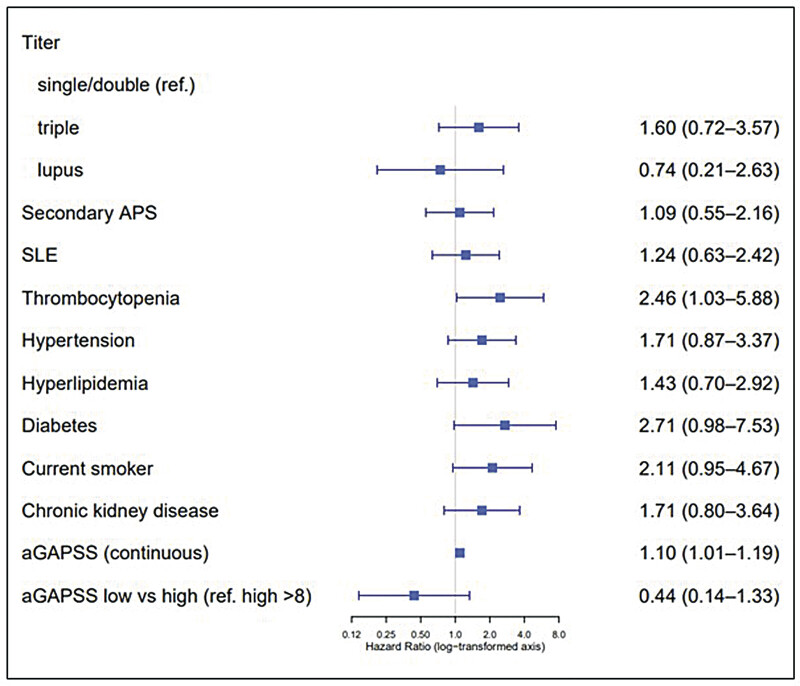
Forest plot presenting the association between cardiovascular and APS-related risk factors and aGAPSS with recurrent thrombosis. Forest plot illustrating the studied cardiovascular and APS-related risk factors associated with recurrent thrombosis. The adjusted Global Anti-Phospholipid Syndrome Score (aGAPSS) is included as a risk factor in the analysis. As shown in the plot, thrombocytopenia and aGAPSS are identified as significant contributors among the evaluated risk factors. The analyses are based on separate Cox regression models, and the reference group for each comparison is now clearly indicated. Additionally, hazard ratios and 95% confidence intervals have been transformed to a log scale for improved visual balance. We have also updated the figure legend to include a full explanation of all acronyms used. Thrombocytopenia was defined as a platelet count <130 × 10 ^9^ /L. Chronic kidney disease (CKD) was defined as an estimated glomerular filtration rate (eGFR) <60 mL/min/1.73 m ^2^ . APS, antiphospholipid syndrome; SLE, systemic lupus erythematosus.

The duration of APS diagnosis before study inclusion was not associated with recurrent thrombosis (HR 1.04, 95% CI 0.98–1.10). Neither secondary APS nor SLE had any effect on the risk for recurrence ( Fig. 2 ).

#### Serological Risk Profile on Antiphospholipid Antibodies

Of the 36 patients with recurrent thrombosis, the most common aPL profile was triple positivity, found in 24 (67%) patients, followed by double positivity in 8 (22%) and only LA in 4 (11%; Table 1 ). Over time, patients with triple positivity showed an increased hazard for recurrent thrombosis compared with those with single and double positivity (HR 1.6, 95% CI 0.72–3.57; Fig. 2 ). Although this was not significant, further analysis revealed a significant association with an increased probability for recurrence (OR 2.52, 95% CI 1.11–6.30). In contrast, no association was observed for recurrent thrombosis in patients with the positivity of LA ( Fig. 2 ).

#### Thrombocytopenia

Of the 20 patients (8%) with thrombocytopenia at inclusion, 5 had recurrent thrombosis during the follow-up ( Table 1 ). There was a significant association between thrombocytopenia and recurrent thrombosis ( Fig. 2 ; HR 2.46, 95% CI 1.03–5.88), which remained after adjustment for sex and age. However, the time-varying Cox model did not show a significant association between thrombocytopenia at multiple time points and risk (HR 2.17, 95% CI 0.89–5.28). Thrombocytopenia also demonstrated a significant difference in IR between patients with and without recurrent thrombosis, with an IRR of 2.23 (95% CI 0.97–4.40), *p* -value = −0.038. Although the *p* -value indicates statistical significance, the CI crosses unity, which may reflect the limited number of patients in this subgroup.

### Cardiovascular Risk Factors

Hypertension, hyperlipidemia, diabetes mellitus, and current smoking were similarly distributed between the two subgroups of patients ( Table 1 ). During the study period, all CV risk factors appeared to increase the risk for recurrent thrombosis; however, none reached statistical significance ( Fig. 2 ). Comparisons of IRs showed that diabetes was significantly associated with the number of recurrent thrombotic events, with a 2.76-fold higher IR (95% CI 1.05–6.01) compared with individuals without diabetes, after accounting for follow-up time.

#### Chronic Kidney Disease

The median eGFR was lower in patients with recurrent thrombosis which corresponded to a significantly higher proportion of CKD in this subgroup compared with those without recurrent thrombosis, 33% and 15%, respectively ( Table 1 ). Patients with CKD had an increased probability for recurrence with an OR of 2.55, 95% CI 1.01–6.26 (adjustments included age, sex, and SLE). However, over time, the association between CKD and recurrent events was not significant (HR 1.71, 95% CI 0.80–3.64; Fig. 2 ). This remained nonsignificant also in a time-varying Cox model, using CKD from multiple time points (data not shown).

### Adjusted Global Anti-Phospholipid Syndrome Score

**Fig. 3:**
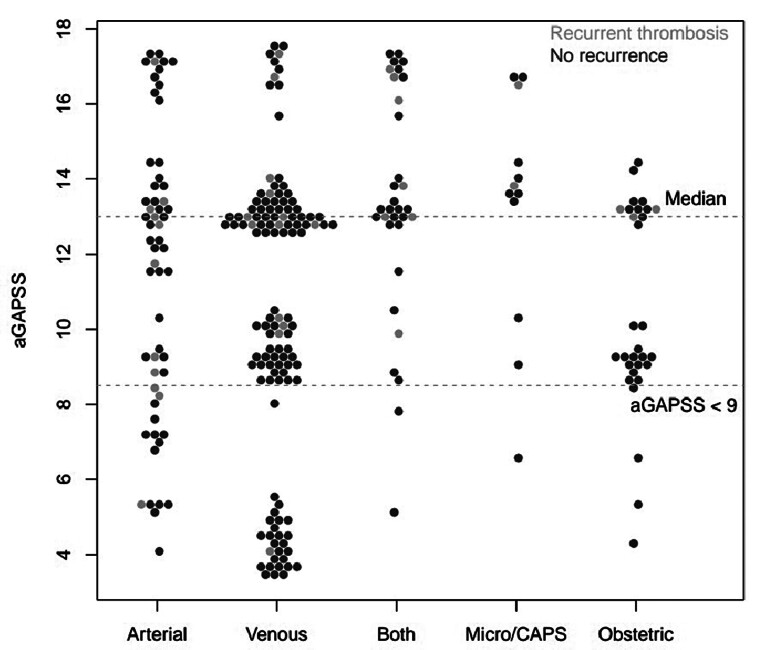
aGAPSS by thrombotic/obstetric APS manifestations at inclusion. Bee swarm plot of aGAPSS APS thrombotic and obstetric manifestations at inclusion, divided into those with recurrent thrombosis and no recurrence. Median score was 13 points, low-risk group was considered as less than 9 points. aGAPSS; adjusted Global Antiphospholipid Syndrome Score; APS, antiphospholipid syndrome; CAPS, catastrophic antiphospholipid syndrome; micro, microthrombosis.

At inclusion, the median aGAPSS was 13 (9–13) points for the entire cohort. The median score differed between patients with and without recurrent thrombosis at follow-up ( Table 1 ). For each point of increment in the aGAPSS, the risk for recurrent thrombosis increased by 10% ( Fig. 2 ) and remained significant after adjustments with an HR of 1.10, 95% CI 1.01–1.19. Distribution of aGAPSS by type of thrombotic/obstetrics manifestation at inclusion is visualized as a Bee Swarm ( Fig. 3 ). A low risk for recurrent thrombosis was considered if aGAPSS was less than 9 points. However, this assumption could not be confirmed in further analysis ( Table 1 and Fig. 2 ); low versus high aGAPSS with an HR of 0.44, 95% CI 0.14–1.33.

### Antithrombotic Treatment and Triggers for Recurrent Thrombosis

Table 2 provides a descriptive overview of all 36 patients who experienced recurrent thrombosis, including clinical context and potential triggers identified at the time of events. Ten of these patients suffered two to three recurrent events during the study period. Seven (14%) recurrent events, including five ATE and two VTE, occurred in patients without any ongoing antithrombotic treatment. Four patients had recurrent events while on direct oral anticoagulants (DOACs), three were treated with Warfarin with a subtherapeutic international normalised ratio, and two patients received prophylactic doses of low-molecular-weight heparin (LMWH). Additionally, two patients suffered from ATE while on platelet inhibitors. The only patient experiencing catastrophic antiphospholipid syndrome (CAPS) as a recurrent event was solely on a platelet inhibitor. In total, 11 (23%) recurrent events were identified on insufficient antithrombotic treatment according to European League against Rheumatology recommendations. 7 Other plausible triggers for recurrence included infection, flare of APS/SLE, surgery, and vascular procedures. Eight recurrent thromboses occurred in patients treated with either Warfarin with documented therapeutic INR or LMWH without any documented trigger in the medical records.

**Table 2:** Descriptive table on recurrent thrombosis and clinical characteristics at inclusion

Clinical characteristics at inclusion						Description on recurrent thrombosis, treatment and plausible trigger		
	Sex	Age	aPL	aGAPSS	APS manifestation	Localization	Antithrombotic treatment	Trigger
1	Male	70	Triple	17	A + V	MI	Warfarin	Vascular procedure
2	Male	71	Triple	17	A + CAPS + V	A, arm	Warfarin + Aspirin	Not identified
3	Male	62	Triple	17	CAPS	TIA	Warfarin + Aspirin	Not identified
4	Female	47	Triple	17	V	A, retinal	Warfarin	Onset of SLE
5	Female	77	Triple	17	A + V	A, leg	None	No treatment
6	Male	48	Triple	17	V	DVT	LMWH	Infection; severe COVID-19
7 (1)	Female	59	Triple	16	A + O + V	MI	Warfarin	Not identified
7 (2)	Female	59	Triple	16	A + O + V	PE	Warfarin	Monitoring issues, large INR fluctuations
8 (1)	Female	62	Triple	14	O + V	IS	Warfarin	Not identified
8 (2)	Female	62	Triple	14	O + V	IS	DOAC	Recent switch from Warfarin
8 (3)	Female	62	Triple	14	O + V	IS	Prophylactic LMWH	Subtherapeutic dose
9	Female	43	Triple	14	A + CAPS + O	A, leg	Warfarin	Vascular procedure
10	Female	36	Triple	14	A + O	DVT	Aspirin	Insufficient antithrombotic treatment
11	Male	43	Triple	14	A + V	TIA	Warfarin	Carotid artery dissection
12	Female	67	Triple	13	A	Micro	Warfarin + Aspirin	Vascular disease/ulcer
13	Female	58	Triple	13	A + O	MI	DOAC	Insufficient antithrombotic treatment
14 (1)	Female	47	Triple	13	O + V	V, stent	Warfarin	Vascular procedure
14 (2)	Female	47	Triple	13	O + V	PE	Warfarin	Vascular procedure
15	Female	43	Triple	13	A + O + V	MI	Warfarin + Aspirin	Not identified
16	Female	42	Triple	13	V	MI	Warfarin	Not identified
17	Male	35	Triple	13	V	PE	None	No treatment
18 (1)	Female	27	Triple	13	V	DVT	Warfarin	Not identified
18 (2)	Female	27	Triple	13	V	Micro	LMWH	APS/SLE flare
18 (3)	Female	27	Triple	13	V	DVT	LMWH	Active APS
19	Female	24	Triple	13	V	DVT	LMWH	Not identified
20	Male	26	Triple	13	A	Heart valve	Aspirin	Insufficient antithrombotic treatment
21	Female	23	Triple	13	A + Micro	A, arm	Warfarin + Aspirin	Non-compliance to treatment
22	Female	18	Triple	13	A	A, leg	Warfarin	Vascular procedure
23 (1)	Male	18	Triple	13	V	DVT	Warfarin	Subtherapeutic INR
23 (2)	Male	18	Triple	13	V	DVT	Heparin	Active APS
24	Female	39	Triple	13	V	DVT	Warfarin	Subtherapeutic INR
25 (1)	Female	39	Double	13	O + V	IS	Warfarin	Insufficient info. in medical records
25 (2)	Female	39	Double	13	O + V	V, retinal	Warfarin	Not identified
26	Male	65	Double	13	A + V	Portal vein	LMWH	Surgery; Kidney tumor
27 (1)	Male	65	Double	12	A	MI	None	No treatment
27 (2)	Male	65	Double	12	A	MI	None	No treatment
28	Male	76	Double	10	V	PE	DOAC	Infection; pneumonia
29 (1)	Female	43	Double	10	O + V	IS	DOAC	Insufficient antithrombotic treatment
29 (2)	Female	43	Double	10	O + V	Thrombophleb. a	Warfarin	Not identified
30	Female	37	Double	10	V	DVT	Prophylactic LMWH	Surgery; subtherapeutic dose
31	Female	80	Double	9	A	IS	None	No treatment
32 (1)	Female	35	Double	9	A	IS	Warfarin + Aspirin	Vertebral artery dissection
32 (2)	Female	35	Double	9	A	V, arm	Warfarin	Not identified
32 (3)	Female	35	Double	9	A	IS	Warfarin	Subtherapeutic INR
33	Female	52	LAC only	8	A	V, arm	Warfarin	Not identified
34 (1)	Male	39	LAC only	8	A	IS	None	No treatment
34 (2)	Male	39	LAC only	8	A	IS	Aspirin	Inflammation; Behcet's disease
35	Female	45	LAC only	5	A	TIA	Aspirin	Insufficient antithrombotic treatment
36	Male	31	LAC only	4	V	PE	None	No treatment

Abbreviations: A, arterial thrombosis; aGAPSS, adjusted global APS score; aPL, antiphospholipid antibody; CA, coronary angiography; CAPS, catastrophic antiphospholipid syndrome; DVT, deep vein thrombosis; IS, ischemic stroke; LMWH, low-molecular-weight heparin; MI, myocardial infarction; Micro, microthrombosis; O, obstetric; PE, pulmonary embolism; TIA, transitory ischemic attack; V, venous thrombosis.

a Extensive thrombophlebitis to the leg on Warfarin treatment that required LMWH in addition.

### Mortality and Cause of Death

Ten deaths occurred during the follow-up time at a median age of 67 years (IQR 52.2–71.5) with an estimated mortality rate of 0.75 per 100 person-years (95% CI 0.4–1.4), which represented a cumulative mortality of approximately 4%. The main cause of death was bacterial infection with sepsis, occurring in three patients. Two patients died from cancer. The remaining four patients died due to pulmonary edema, carotid dissection, MI, and respiratory failure secondary to COVID-19. In one patient, the cause of death could not be identified. Among patients who died, there were as many patients with primary as secondary APS.

## Discussion

This single-center cohort of 250 APS patients provides valuable insights into the impact of both established CV and APS-related risk factors on recurrent thrombotic events. During the 5-year follow-up, 49 recurrent thrombotic events occurred in 36 (14%) patients, whereas 10 patients suffered two to three recurrent events. We confirmed that mild thrombocytopenia and an increase in aGAPSS were robustly associated with recurrent thrombosis in APS. None of the CV risk factors was consistently significant for recurrent thrombosis; however, our study identified CKD as a potential contributing risk factor. Notably, the absence of adequate anticoagulant treatment was a substantial trigger for recurrent thrombosis.

Recurrent thrombosis occurred in 14% of the patients during the follow-up, a proportion similar to the 17% found in the largest international APS cohort with 1,000 patients. 4 Other studies have reported as high as a 40% recurrence rate, explained by the heterogeneity between studies, that is, differences in the studied population or anticoagulation treatment at the time of recurrence. 1 5 Strikingly, 10 patients in our cohort suffered two or more repeated events, resulting in a substantial morbidity in this subgroup. Women were overrepresented in our study population and were diagnosed approximately 10 years before men, even if patients with only obstetric manifestations were excluded. Nonetheless, we found no impact of age, sex, or disease duration at inclusion on the risk for recurrent thrombosis. Patients with secondary APS (38%) were not affected by recurrent thrombosis to a greater extent than those with primary APS.

The prediction of recurrent thrombosis in APS patients remains a clinical challenge. There are ongoing efforts to improve assessment of patients at highest risk, including novel classification criteria with thrombocytopenia, microangiopathy, and cardiac valve disease as important clinical domains for APS classification. 22 The initiative by the APS ACTION group introducing aGAPSS is also of importance and has so far been validated in different APS clinical settings. 8 11 23 24 25 26 However, in daily clinical practice, the aPL profile is considered the most useful tool when determining the risk of recurrence and thereby the intensity of treatment.

We confirmed that the serological high-risk profile with triple positivity for aPL was associated with the probability of suffering a recurrent thrombotic event. Among traditional CV risk factors, only diabetes mellitus showed a significant association with recurrent thrombotic events in additional analyses. Notably, when hypertension and hyperlipidemia were combined into the aGAPSS, a significant association with recurrent thrombosis was shown. For each additional point in aGAPSS, the hazard for recurrent thrombosis increased by ten percent. These findings support the concept of the “second hit” hypothesis for thrombosis development in APS, where the presence of aPL is necessary but additional factors like uncontrolled traditional CV risk factors are also required to shift the hemostatic balance toward a procoagulant state. 26 The aGAPSS incorporates both serological APS features and CV risk factors into the risk prediction model and may serve as a useful and easily accessible tool for clinicians. Nevertheless, aGAPSS still needs to be validated in prospective cohorts. Ideally, a cutoff between high and low risk for recurrence could then be identified, an attempt we were unable to achieve.

The mechanisms leading to aPL-induced thrombocytopenia are complex. Platelet activation and consumption occur due to interaction of immune complexes with platelet membrane receptors, like aβ _2_ -GP1–β _2_ GP1 interaction with GPIIb/IIIa or through direct antibodies against glycoproteins on the cell membrane of platelets. 15 27 28 Although it remains uncertain whether these antibodies can be used in the prognostic evaluation of APS patients, several studies have indicated that thrombocytopenia can serve as a warning sign for assessing high-risk APS. 16 The prevalence of thrombocytopenia in our study was 8% and it was significantly associated with a risk for recurrent thrombosis. In previous APS cohorts, the prevalence of thrombocytopenia ranged from 15 to 40% 29 30 31 and the estimated bleeding risk associated with it was lower than the thrombotic risk. 16 In addition, in the subgroup with recurrent events, 14% (5/36) had thrombocytopenia and these patients suffered from severe clinical presentations, one even with CAPS during the follow-up period. A limited number of earlier studies have investigated the relation between low platelet count and clinical APS manifestations. Thrombocytopenia has been shown to be an independent risk factor for thrombosis in aPL carriers 32 33 34 and is especially associated with arterial thrombosis. 30 According to Sun et al., it is likely that continuous low-grade activation of platelets by cofactor–antibody complexes leads to increased platelet turnover, eventually causing thrombosis in combination with thrombocytopenia. 35 This idea supports the proposal of a positive feedback loop between thrombocytopenia and the formation of platelet-rich thrombi.

Impairment of kidney function may be relevant in predicting the risk for recurrent thrombosis, as it could indicate more active and/or severe APS disease. Renal involvement in APS has been found in 10 to 40%. 36 In our study, the prevalence of CKD was 18%, which is very similar to a recently published large Taiwanese population study on APS. 37 Our additional analysis found that CKD increased the likelihood of recurrent thrombosis even after adjusting for SLE. Out of the patients with CKD 48% had SLE, while the rest of the patients had primary APS, showing no difference between these two subgroups of patients regarding the occurrence of CKD. It should be noted, however, that our study used only the eGFR as an indicator of kidney function, and we lack data from kidney biopsies. According to previous results by our group, histopathological findings consistent with thrombotic microangiopathy associated with aPL were common among SLE patients with renal involvement, being present in around 15% of these patients. 38 Furthermore, patients with biopsy-confirmed, APS-associated nephropathy had worse kidney function compared with those with lupus nephritis alone. 38 These findings emphasize the importance of continuous monitoring of kidney function in APS patients.

A possible trigger of recurrent thrombosis could be determined in most of the patients. A majority of these were treatment-related, including patients being treated with DOACs, warfarin compliance issues, sole treatment with platelet inhibitors, recent switch from warfarin to LMWH, or not being treated at all. Four patients had also recently undergone vascular procedures and surgery. Taken together, almost one-fourth of all recurrent events occurred in patients with insufficient antithrombotic treatment. These results are in-line with the outcomes of previous studies, indicating the importance of adequate anticoagulation and treatment monitoring in APS patients. 7 39 40

Patients in our cohort had a mortality rate of 4% during the follow-up and deaths occurred at relatively young age, similar to patients in our SLE cohort. 41 Of note, the presence of any aPL among our SLE patients was predictive for CV-related mortality. Thus, intensive monitoring and treatment of CV risk factors and disease-related risk factors for recurrent thrombosis are of utmost importance in patients with APS.

The major limitation of this study is the retrospective design, which might introduce biases related to data availability and accuracy. Since the single-center scope was applied, findings may not be fully generalizable to other settings. However, this setting has advantages since data were collected by two experienced specialists to limit the false data interpretation. The focus on a single referral hospital for APS in Stockholm ensured consistency in data quality. Taking into consideration the rarity of this syndrome, we have included a large number of well-characterized APS patients during the 5-year follow-up period. An important strength of our study is the standardized approach to laboratory analyses, including aPL assessment. Notably, we employed a solid-phase chemiluminescence immunoassay method, which, although well-validated for APS and routinely used at Karolinska University Laboratory since 2014, differs from the ELISA-based assays recommended by the updated Sapporo criteria. 18 19 20 This may limit direct comparability with studies using ELISA. 19 20 Finally, given the relatively small number of events in our cohort, we supplemented the Cox regression analysis with exploratory logistic and Poisson regression models. We acknowledge the methodological limitations of this approach, particularly the use of logistic regression in a cohort with a heterogenous follow-up duration. The Poisson regression was applied to explore IR differences, although it was not predefined in the study design. These secondary analyses were included to offer additional perspectives and are interpreted with caution.

## Conclusion

Predicting the risk of thrombosis recurrence in APS in an individual patient remains a challenge. Our study confirms that an increased aGAPSS, which includes both serological and CV risk factors, is associated with recurrent thrombosis, although prospective studies are necessary to further refine its clinical application. Moreover, thrombocytopenia, recently included in the new APS classification, was found to be significantly associated with recurrent thrombosis, supporting the potential relevance in risk stratification. 19 Impaired kidney function also emerged as an additional factor that warrants closer attention in future risk assessments. Finally, inadequate anticoagulation was the most common trigger for recurrent thrombosis, emphasizing the critical need for improved surveillance and management of treatment in APS patients.

## Notes

**What Is Already Known on This Topic?**

- Recurrent thrombosis is common in APS patients and poses a significant treatment challenge. Predicting the risk of recurrence for the individual patient remains difficult.

**What Does This Study Add?**

- In addition to confirming that mild thrombocytopenia and an elevated aGAPSS were associated with recurrent thrombosis in APS, our study identified CKD as a potential contributing risk factor.

**How Might This Study Affect Research, Practice, or Policy?**

- The main trigger for recurrent thrombosis in our cohort was inadequate anticoagulant treatment, which highlights the need for increased awareness and closer surveillance of treatment in APS patients.
